# Reading Notice

**Published:** 1906-07

**Authors:** 


### READING NOTICE.

An Old and yet a New Antiseptic.—Campho-Phénique, although on the market for the last twenty-five years, is only being generally used by dentists for Dental Surgery, during the past few years, and it is accepted by all the best dentists, to be a great improvement over all other antiseptics, where indicated. The Campho-Phénique Company will be glad to supply samples, or give any further information, upon receipt of a request.

If you want a loction in Western or Middle States address J. Franklin Steele, D. D. S., Eagle Grove, Iowa.

For the Public Good.—An independent and ably edited newspaper which commands a great circulation is probably the most potent influence for good in the United States today. The power for the better things in public affairs and policies, for instance, which is wielded by such a newspaper as The Chicago Record-Herald can scarcely be exaggerated, and much of that strength comes in the case of this leading Chicago daily from the fact that it is absolutely independent, fearless and fair. It is not the mouthpiece of any interest except that the public. The Record-Herald champions the cause of the good, the clean, the benefical in every matter of city, state or national moment. It is the knowledge on the part of its readers that it cares not whom it hits or what enemies it makes, so long as it is battling for the welfare of the community, which gives to The Record-Herald much of the influence it enjoys. It gives in its news columns the most complete events, another evidence of its splendid news service.

Dr. Bridge of Boston, Mass., has come forward with an Obtunder in which he successfully uses 70 per cent. Alcohol for obunding sensitive dentine. With this agent as a substitute for cocain, it would seem that the day had come for the most conservative Dentist to adopt this all important help of pressure anæsthesic in those severe caces of cavity preparation, grinding for crowns and derilizing which we all meet and dread.

Alcohol at 70 per cent. strength is claimed to be non-irritating and a more potent antiseptic than commercial or absolute, and with pressure as the obtunding factor, this would seem an ideal agent. An adr. appears for Dr. Bridge's Obtunder in this issue.

